# Association of Generation and Group Size With the Usage of a Mobile Health App in Thailand: Secondary Analysis of the ThaiSook Cohort Study

**DOI:** 10.2196/45374

**Published:** 2023-08-17

**Authors:** Tharoj Inchusri, Decho Surangsrirat, Papichaya Kwanmuang, Prapasiri Poomivanichakij, Ponnapat Apiwatgaroon, Surathep Ongprakobkul, Apissara Kongchu, Anda Klinpikul, Ammarin Taneeheng, Nannapat Pruphetkaew, Therdpong Thongseiratch, Pitchayanont Ngamchaliew, Polathep Vichitkunakorn

**Affiliations:** 1 School of Medicine and Health Sciences Faculty of Medicine Prince of Songkla University Songkhla Thailand; 2 Assistive Technology and Medical Devices Research Center National Science and Technology Development Agency Bangkok Thailand; 3 Department of Epidemiology Faculty of Medicine Prince of Songkla University Songkhla Thailand; 4 Division of Developmental and Behavioral Pediatrics, Department of Pediatrics Faculty of Medicine Prince of Songkla University Songkhla Thailand; 5 Department of Family and Preventive Medicine Faculty of Medicine Prince of Songkla University Songkhla Thailand

**Keywords:** application, cohort, generation group size, health care personnel, logging function, mobile health technology, Thailand

## Abstract

**Background:**

In Thailand, The National Science and Technology Development Agency developed ThaiSook, a behavior-tracking app, to promote healthy lifestyles. The Faculty of Medicine, Prince of Songkla University (MED PSU)×ThaiSook Healthier Challenge encouraged employees to use the app over a 28-day period (from July 11 to August 7, 2022). Until recently, no previous studies have examined the association of generations and group sizes with mobile health (mHealth) app use. Understanding these relationships can inform the design of effective mHealth interventions and facilitate targeted interventions.

**Objective:**

This study aimed to (1) compare the overall app usage and logging function across different generations and group sizes and (2) describe the demographic characteristics of the participants of the MED PSU×ThaiSook Healthier Challenge.

**Methods:**

We conducted a secondary data analysis of the data from the ThaiSook prospective cohort study. Data were collected through the app and comprised demographic characteristics (ie, age, sex, weight, height, and group size) and behaviors (ie, water consumption, fruit and vegetable consumption, sleep hours, and exercise). The outcomes consisted of users who used the app for at least 80% of the participation period (≥23 days). Bivariate tests (Pearson chi-square test for categorical variables and Mann-Whitney *U* and Kruskal-Wallis tests for continuous variables) were conducted over sex, generations, initial BMI, and group size. Finally, multiple logistic regression models were used to examine the relationship between the independent variables used by the ThaiSook app and consistent users who had used the app for at least 80% of the participation period.

**Results:**

Of the 827 participants, most were female (734/827, 88.8%), belonged to a medium-sized group of 6-10 members (479/827, 57.9%), and belonged to generation Y (377/761, 49.5%). Multivariate analysis revealed that the overall app usage was 2.09 times higher in women than in men (adjusted odds ratio [AOR] 2.09, 95% CI 1.27-3.44). The older generations used all logging functions more frequently than did generation Y (baby boomers AOR 2.54, 95% CI 1.31-4.92; generation X AOR 1.96, 95% CI 1.42-2.72). The use of all logging functions was higher among participants belonging to larger groups than among those belonging to smaller groups (large groups AOR 2.85, 95% CI 1.58-5.16; medium groups AOR 2.06, 95% CI 1.47-2.88). Water logging was most used (806/827, 97.5% participants), followed by food, sleep, and workout logging.

**Conclusions:**

The MED PSU×ThaiSook Healthier Challenge participants were mostly females from generation Y and medium-sized groups. Water logging was most frequently used, followed by fruit and vegetable logging. The results indicate that generation and group size were significantly associated with consistent and daily usage (*P*<.05). Older generations and larger groups engaged with the app more consistently than younger generations and smaller groups and individuals.

## Introduction

Globally, mobile health (mHealth) technology has grown rapidly in recent years. The global mHealth app market size was expected to grow at a compound annual growth rate of 25.3% in 2021-2022. mHealth technology mainly targets healthy individuals for daily use and fitness activities. However, another target group comprises patients with noncommunicable diseases who use mHealth apps for health monitoring [1]. For chronic diseases such as hypertension [2-5] and diabetes [6-8], mHealth technology enables changes in health risk behavior, an improvement in patient compliance, and an increase in self-efficacy [9]. At the forefront of mHealth are mHealth apps, which leverage the widespread use of mobile devices to deliver health care services, monitor health conditions, and promote healthy lifestyles [10]. These apps offer a range of features and functionality, including tracking health behaviors, access to health care, remote patient monitoring, health education, and personal health management [11]. They have the potential to improve health care access, enhance patient engagement, and empower individuals to take control of their health. mHealth tracking apps are among the most common types of mHealth apps [12], which allow users to monitor and track various health parameters such as physical activity, nutrition, sleep patterns, and vital signs, providing individuals with insights into their lifestyle habits and offering opportunities for behavior change and improvement. By empowering users to take control of their health, these apps have gained significant popularity in the mHealth landscape. Notably, the Asia-Pacific region is the fastest-growing market for mHealth apps [13-15]. In Thailand, 66.8% of the general population used health and wellness apps in 2020. “Sports and fitness activities” and “diet and nutrition” were the 2 most frequently used functions [15].

Factors influencing the frequent use of mHealth tracking apps include sex [12,16], education [12,17], technological literacy [17], peer influence [18], performance expectancy, social influence, and facilitating conditions [19]. On closer examination, a person’s generation is one of the factors that could influence the use of mHealth apps, in part because different generations have different exposure to technology. baby boomers, generation X, millennials (generation Y), and generation Z are distinct generational categories based on birth years and shared cultural experiences. Furthermore, previous studies and surveys showed that each generation prefers to use mHealth apps and wearable devices, with most members of generation Z reporting using mHealth apps at least once in their lifetime due to growing up in a highly digital environment, which makes them comfortable with technology [20,21]. On the other hand, generation Y uses considerably more wearable health devices than do the other generations [22], and generation X individuals show moderate adoption rates of mHealth tracking apps as they tend to be more open to using these apps compared to baby boomers but may still lag behind generation Y in terms of adoption. Baby boomers generally have lower adoption rates of mHealth tracking apps compared to younger generations. Factors such as lower technological familiarity, concerns about privacy and security, and perceived complexity of app usage might be identified as baby boomers’ adoption barriers. This suggests that different generations have different levels of confidence in the usefulness of mHealth technology in improving health. Another factor found to influence the use of mHealth apps is group size. Larger groups increase member attraction, leading to more cooperative outcomes [23]. Furthermore, group size also influences group function, member attachment, and stability [24].

ThaiSook, the mHealth app developed by the National Science and Technology Development Agency (NSTDA), aimed to reduce health-risk behaviors and promote a healthy lifestyle. Participants used the app to improve their desirable health behaviors through a web-based competition model with expert coaching, team building, and group competition to win rewards. Predictors of premature death were recorded, namely weight, vegetable and fruit consumption, exercise, blood pressure, blood lipid levels, blood sugar levels, and smoking. These 7 parameters were based on Folsom’s publication, “American Heart Association’s Life’s Simple 7: Avoiding Heart Failure and Preserving Cardiac Structure and Function.” According to Life's Simple 7, the aforementioned 7 predictors highly associated with premature death [25,26] were recorded in ThaiSook. ThaiSook is a self-recorded program in which users can record data such as weight, laboratory tests, and behaviors (ie, water consumption, fruit and vegetable consumption, and sleep logging). In addition, the app can connect with fitness tracking (ie, Apple Health, Google Fit, and Xiaomi Scale 2) and automatically retrieve information, such as step or run logging. The Wellness Center of the Health District System adopted ThaiSook in 63 hospitals across the country and in other health-promoting offices to expand the app’s usage to a larger population.

To date, no research has explored the association between generations, group sizes, and the use of mHealth apps. Therefore, further research is warranted to comprehensively comprehend the intricate interplay between these factors. Gaining insights into these dynamics can inform the development of effective mHealth interventions and facilitate targeted approaches. In 2022, a collaboration between the NSTDA and the Faculty of Medicine of the Prince of Songkla University (MED PSU) led to the MED PSU×ThaiSook Healthier Challenge. This initiative encouraged employees to use the app over a 28-day period, presenting an opportunity to address this knowledge gap through research. Consequently, this study aimed to (1) compare the overall usage of the app and the logging function across different generations and group sizes and (2) describe the demographic characteristics of the participants in the MED PSU×ThaiSook Healthier Challenge.

*Hypothesis 1 (H1):* A person’s generation positively associates with mHealth usage and logging function.

*Hypothesis 2 (H2):* Group size positively associates with mHealth usage and logging function.

## Methods

### Study Design and Study Setting

This study was a secondary analysis of data from the NSTDA’s prospective cohort study (ie, MED PSU×ThaiSook Healthier Challenge), which in turn was extracted from the ThaiSook database.

### MED PSU×ThaiSook Healthier Challenge

The MED PSU×ThaiSook Healthier Challenge, a collaboration between NSTDA, who developed the ThaiSook app, and the Faculty of Medicine of the Prince of Songkla University (PSU), encouraged employees to use the app over a 28-day period. The campaign aimed to promote employees’ healthy behaviors in the Faculty of Medicine.

#### Announcement and Invitation Period

One week before the kickoff event, communication channels such as circular letters, internal messaging platforms, bulletin boards, and web-based newsletters were used to raise employee awareness about the campaign. The Faculty of Medicine’s website also featured a dedicated section providing detailed information about the campaign, its goals, and how to participate.

#### Kickoff Event

The kickoff event for the mHealth tracking campaign, named “MED PSU×ThaiSook Healthier Challenge,” took place on July 6, 2022, from 9 AM to 12 PM. The event was held in the multipurpose area of the Faculty of Medicine. The event was also live-streamed and recorded through the faculty’s Facebook account. To set the tone for the campaign and inspire employees, ThaiSook’s developer from NSTDA (DS) and 4 physicians from the faculty (ie, epidemiologist [PV], cardiologist, clinical nutritionist, and rehabilitation doctors) delivered the keynote address. The developer guided the employees through the app’s features, functionality, and data input process. This demonstration showed how to track activities, monitor progress, and use the app’s resources. To ensure that employees were proficient in using ThaiSook, interactive training workshops were conducted. These sessions were led by the developer and trained facilitators who were knowledgeable about the app’s features and functionality. During the training sessions, employees were provided with their smartphones preloaded with ThaiSook. This hands-on approach allowed participants to navigate the app, explore its features, and practice data input and tracking.

#### Challenge Period

The MED PSU×ThaiSook Healthier Challenge was held for 28 days (from July 11 to August 7, 2022) at the Faculty of Medicine (PSU). Of the 6112 employees, 827 participants (a response rate of 13.5%) were divided into 164 teams of different sizes and took part in the challenge. They were allowed to form teams independently, with no limit on the number of group members; thus, even 1-person teams were included. All the data were self-reported (see details in the “Data Collection” section). The participants submitted their date of birth and self-recorded their weight, height, and health behavior findings. Furthermore, they were encouraged to track their health parameters regularly using ThaiSook. The app provided real-time personalized insights based on the tracked data. Real-time progression reports were generated and shared with participants in the group.

### Participants

To meet inclusion criteria, employees (ie, health care and non–health care personnel) had to be employees of the Faculty of Medicine, PSU, who participated in the ThaiSook between July 11, 2022, and August 7, 2022. The exclusion criterion was the following: participants who had not reported their birth date in the ThaiSook.

### Power Analysis

Since there have been no previous studies on the association between the use of the app (ie, dependent variable) across different generations and group sizes (ie, primary independent variables), a power analysis was conducted.

A post hoc power analysis was conducted to assess the achieved power in logistic regression. The analysis used odds ratios of 2.54 (95% CI 1.30-4.92); 1.96 (95% CI 1.42-2.72); 0.24 (95% CI 0.07-0.84); 2.06 (95% CI 1.47-2.88); and 2.85 (95% CI 1.58-5.16) for baby boomers, generation X, and individual, medium, and large groups, respectively. The hypothesized probability of Y=1 given X=1 was set at *P*=.61, *P*=.58, *P*=.17, *P*=.56, and *P*=.59 for baby boomers, generation X, individual, medium, and large members, respectively. The X parameter π was set at 0.061; 0.360; 0.029; 0.579; and 0.079, respectively. A significance level (α) of .05 and a total sample size of 761 were applied. The power analysis yielded a power of 0.7; 1; 0.8; 1; and 0.9, respectively, for all variables. These results indicate sufficient power to detect the anticipated effect size with the given sample size and significance level for the logistic regression model, except for the baby boomer group.

### Data Collection

**Figure 1:**
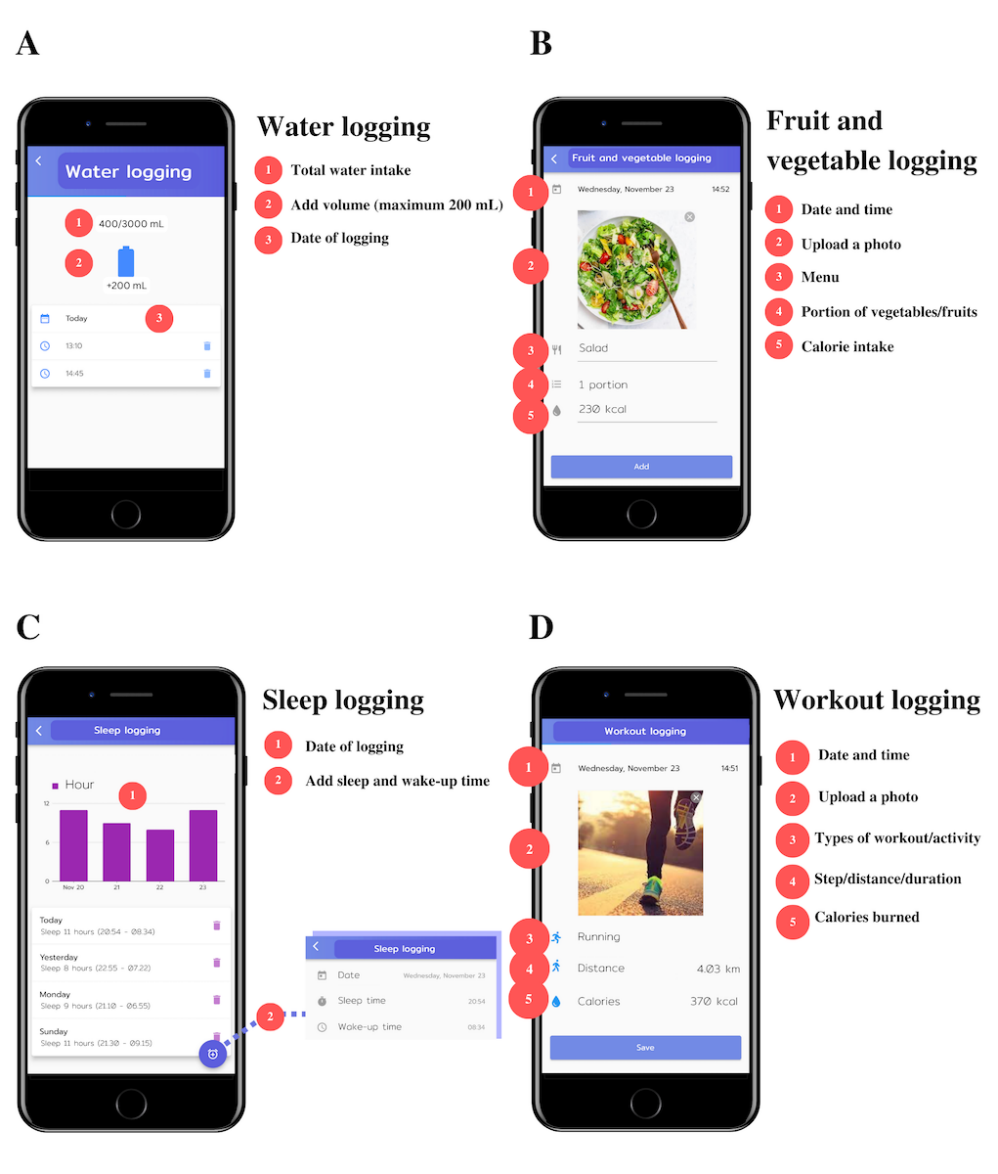
An example of logging functions (A) water logging, (B) fruit and vegetable logging, (C) sleep logging, and (D) workout logging in the ThaiSook app.

The data used were secondary data from the MED PSU×ThaiSook Challenge, which belonged to the NSTDA and the Faculty of Medicine of PSU. The data were recorded by the participants. All collected data were kept confidential and were only accessible to the researchers through the ThaiSook database. The data consisted of 2 parts: the demographic characteristics of the participants (age, sex, weight, height, and group size) and the use of the ThaiSook app (logging functions; Table 1 and Figure 1).

**Table 1:** Details of all logging functions used in the ThaiSook app.

Logging functions		Details of each function
App use		Record daily activities using logging functions (ie, exercise, food and water consumption, sleep hours, weight, blood pressure, and blood test results), and the app will then show a summary of usage and overall health behaviors. Moreover, in a web-based challenge, users may participate in a team-based competition.
Water logging		Record date, time, and volume in integer values, with a maximum volume of 200 mL for each log. For example, a drinking water volume of 400 mL would be 2 logs of 200 mL and would be counted as 2 records. The maximum volume recorded for a day was 3000 mL.
Fruit and vegetable logging		Upload a picture of food with the menu and record portions of fruit and vegetables and calories consumed (optional). However, this function can be used to record meals without fruits or vegetables.
Sleep logging		Record wake-up time and bedtime; the app will then calculate the total sleep time. The user can record more than once per day.
**Workout logging**		Upload a picture, choose the type of activity (ie, stepping, running or walking, cycling, meditation, and others), and record the calories burned (optional). There are different recording details for each type of activity.
	Step logging	Upload a picture and record the number of steps taken in a day (only the last recorded steps will be logged that day), distance walked (optional), and calories burned (optional).
	Run logging	Upload a picture and record the running duration (minutes), distance (optional), and calories burned (optional).

#### Independent Variables: Group Size and Generation

The primary independent variables were group size and generation. Group size was classified into the following 4 groups: individual (1 member per group), small (2-5 members per group), medium (6-10 members per group), and large (>10 members per group). The participants were divided into the following 4 generations based on the year of their birth: baby boomers (born in 1946-1964; current age range 58-76 years) [27]; generation X (born in 1965-1980; current age range 42-57 years old); generation Y (born in 1981-1996; current age range 26-41 years); and generation Z (born in 1997-2012; current age range 10-25 years) [27,28]. Other independent variables included sex (male or female) and the initial BMI. BMI was calculated as weight in kilograms divided by the square of the height in meters (kg/m^2^); it was classified according to the World Health Organization Asian BMI classifications into the following: underweight (<18.5 kg/m^2^), average (18.5-22.9 kg/m^2^), overweight (23-24.9 kg/m^2^), obese class I (25-29.9 kg/m^2^), and obese class II (≥30 kg/m^2^) [29].

#### Dependent Variable: App Use

The MED PSU×ThaiSook Challenge was conducted for 28 days. The number of days the app was used serves as a measure of user participation. Participants who had used the app for at least 80% of the total participation period (not less than 23 days) were defined as “consistent users,” and those who had used the app every day were defined as “daily users.” Furthermore, we measured the daily usage frequency of the overall app and all logging functions (time and usage per day).

### Statistical Analysis

Descriptive statistics, such as medians with IQRs, were used to express the demographic characteristics and app logging data (water intake, fruit and vegetable consumption, sleep, and workout). The statistical analysis began by conducting a Shapiro-Wilk test to assess the normality of the variables. After confirming the normality assumption, Pearson chi-square test was used to compare categorical variables, and the Mann-Whitney *U* and Kruskal-Wallis tests were used to compare the continuous variables. Multiple logistic regression models were used to measure the magnitude of the independent variables that affected consistent users’ usage of the ThaiSook app. The initial model included potential independent variables (*P*<.20), namely sex, group size, generation, and initial BMI. Univariate analysis yielded several variables (*P*<.20). To derive the final model, a manual backward stepwise refinement process was conducted. Adjusted odds ratios (AORs) along with their corresponding 95% CIs were calculated. Statistical significance was determined at a threshold of *P*<.05. The data analysis was performed using R software (version 4.2.1; R Foundation) and several packages, including epicalc, foreign, survival, MASS, nnet, data.table, rio, and dplyr.

### Ethics Approval

The clinical trial was registered according to the WHO International Clinical Trials Registry Platform (WHO-ICTRP) at the Thai Clinical Trials Registry registry ID TCTR20220611001). This study was approved by the Human Research Ethics Committee of the Faculty of Medicine of the Songkla University (REC.65-395-9-2). The HREC allows additional informed consent to be waived from the secondary analysis. All collected data from the participants were kept confidential, anonymous, and accessible only to the researchers through the ThaiSook database. No compensation for participants was provided.

## Results

### Participant Characteristics and Usage of the ThaiSook App

Overall, 827 individuals participated in this study. Among these, approximately 49.8% (412/827) were deemed consistent users, and one-third were deemed daily users (249/827, 30.1%). The median usage duration was 22 days (IQR 12.0-28.0; Table 2).

**Table 2:** Participant characteristics by usage frequency of ThaiSook app (N=827).

Characteristics			Participants, n (%)		Average usage duration (days), median (IQR)		Consistent users (≥80%), n (%)				Daily users (≥100%), n (%)		
							Yes		No		Yes		No
Total			827 (100)		22 (12-28)		412 (49.8)		415 (50.2)		249 (30.1)		578 (69.9)
**Sex^a^**													
	Male	93 (11.2)		15 (8-26)		29 (31.2)		64 (68.8)		18 (19.4)		75 (80.6)	
	Female	734 (88.8)		23.5 (13-28)		383 (52.2)		351 (47.8)		231 (31.5)		503 (68.5)	
	Total	827		22 (12-28)		412		415		249		578	
**Generation^b^**													
	Baby boomer	46 (6.1)		26.5 (17-28)		28 (60.9)		18 (39.1)		19 (41.3)		27 (58.7)	
	Generation X	274 (36.0)		25 (17-28)		160 (58.4)		114 (41.6)		107 (39.1)		167 (60.9)	
	Generation Y	377 (49.5)		20 (11-27)		161 (42.7)		216 (57.3)		91 (24.1)		286 (75.9)	
	Generation Z	64 (8.4)		18 (8-26)		24 (37.5)		40 (62.5)		9 (14.1)		55 (85.9)	
	Total	761		22 (12-28)		373		388		226		535	
**Initial BMI^c^**													
	Underweight	51 (6.2)		20 (11.5-27)		21 (41.2)		30 (58.8)		11 (21.6)		40 (78.4)	
	Average	376 (45.4)		22 (12-28)		186 (49.5)		190 (50.5)		111 (29.5)		265 (70.5)	
	Overweight	138 (16.7)		24.5 (14-28)		75 (54.3)		63 (45.7)		42 (30.4)		96 (69.6)	
	Obese class I	215 (26.0)		23 (14-28)		112 (52.1)		103 (47.9)		74 (34.4)		141 (65.6)	
	Obese class II	47 (5.7)		19 (11-27)		18 (38.3)		29 (61.7)		11 (23.4)		36 (76.6)	
	Total	827		22 (12-28)		412		415		249		578	
**Group size^d^**													
	Individual (1 member per group)	24 (2.9)		4.5 (2-10.25)		4 (16.7)		20 (83.3)		2 (8.3)		22 (91.7)	
	Small group (2*-*5 members per group)	259 (31.3)		19 (10-27)		102 (39.4)		157 (60.6)		55 (21.2)		204 (78.8)	
	Medium group (6*-*10 members per group)	479 (57.9)		25 (15-28)		268 (55.9)		211 (44.1)		171 (35.7)		308 (64.3)	
	Large group (≥11 members per group)	65 (7.9)		24 (9-28)		38 (58.5)		27 (41.5)		21 (32.3)		44 (67.7)	
	Total	827		22.0 (12-28)		412		415		249		578	

^a^Average usage duration: *P*<.001 (Mann-Whitney *U* test), nonconsistent users: *P*<.001 (Pearson chi-square test), and non–daily users: *P*=.02 (Pearson chi-square test).

^b^Average usage duration: *P*=.002 (Kruskal-Wallis test), nonconsistent users: *P*<.001 (Pearson chi-square test), and non–daily users: *P*<.001 (Pearson chi-square test).

^c^Average usage duration: *P*=.26 (Kruskal-Wallis test), nonconsistent users: *P*=.23 (Pearson chi-square test), and non–daily users: *P*=.32 (Pearson chi-square test).

^d^Average usage duration: *P*=.004 (Kruskal Wallis test), nonconsistent users: *P*<.001 (Pearson chi-square test), and non–daily users: *P*<.001 (Pearson chi-square test).

The participants were predominantly female (734/827, 88.8%), belonged to generation Y (377/761, 49.5%), and had a normal BMI (376/827, 45.4%). The majority of participants were in the medium-sized group (479/827, 57.9%), followed by the small-sized (259/827, 31.3%) and large-sized (65/827, 7.9%) groups. Only 24 users (24/827, 2.9 %) participated individually (1 member per group).

Sex, generation, and group size had a statistically significant impact on the average app usage duration (*P*<.05). The number of participation days was higher for female participants (23.5 days) than for male participants (15 days). The older generations (ie, baby boomers and generation X) used the app more routinely than did the younger generations (ie, generations Y and Z). Moreover, the study results showed that group users had a longer duration of app usage (ranging from 19 to 15 days) than did individual users (4.5 days). In contrast, the initial BMI did not significantly affect the average usage duration.

Furthermore, the study results showed that sex, generation, and group size were significantly associated with consistent and daily usage (*P*<.05). Strikingly, the older generations used the app more regularly than did the younger generations, and participants belonging to larger groups used the app more regularly.

### Average Usage Duration and Daily Usage Frequency of all Logging Functions

The average duration of app use was 22 days, and data were recorded 11.58 times per day (IQR 7.50-14.91; Table 3). The most regularly used logging function was water logging (21 days), followed by fruit and vegetable logging (19 days), sleep logging (19 days), and workout logging (13 days).

**Table 3:** Average usage duration and daily usage frequency of all logging functions (N=827).

Logging function			Participants, n	Average usage duration (days), median (IQR)	Daily usage frequency (times per usage day), median (IQR)	
**App use**			827	22 (12-28)	11.58 (7.50-14.91)	
	Water logging		806	21 (11-18.6)	8.33 (6.43-9.85)	
	Food logging		755	19 (10-26)	2.64 (1.64-7.04)	
	Sleep logging		736	19 (9-24)	1.04 (1-1.15)	
	**Workout logging**		669	13 (5-23)	1.11 (1-1.38)	
		Step logging	448	7 (2-15)	1 (1-1)	
		Run logging	513	7 (3-15)	1 (1-1.08)	

In terms of the daily usage frequency, the study results showed that participants most frequently recorded water logging (8.33 times per usage day), followed by fruit and vegetable logging (2.64 times per usage day). Other logging functions were used approximately once a day (ie, sleep and workout logging).

### Factors Influencing ThaiSook’s Consistent Users

Regarding consistency in app usage, female participants were 2.09 times more likely to be consistent users compared to male participants (AOR 2.09; 95% CI 1.27-3.44; Table 4). With respect to generations, the older generations were more likely to be consistent users as compared to generation Y (baby boomers AOR 2.54, 95% CI 1.3-4.92; generation X AOR 1.96, 95% CI 1.42-2.72). Participants from larger groups were more likely to use the app more consistently compared to those from smaller and medium groups (large groups AOR 2.85, 95% CI 1.58-5.16; smaller groups AOR 2.06, 95% CI 1.47-2.88).

Regarding generation, the use of all logging functions was higher in the older generations than in generation Y (baby boomers AOR 2.02-3.21; generation X AOR 1.59-2.05). The younger generations used logging functions less frequently, especially for water, fruit and vegetable, and sleep logging.

In terms of group size, medium (AOR 1.57-2.51) and large (AOR 2.29-4.19) groups were associated with higher use of all logging functions (water, fruit and vegetable, sleep, and workout logging) as compared to small groups. Compared with small groups, individual (1-member) groups were approximately 4-5 times less likely to use the overall app and water logging.

**Table 4:** Relationship between demographic data and consistent users (≥80%) (n=761).

Variable^a^		Overall App usage consistent users (n=761), AOR^b^ (95% CI)	Consistent users (≥80%) of app function			
			Water consistent users (n=742), AOR (95% CI)	Food consistent users (n=693), AOR (95% CI)	Sleep consistent users (n=677), AOR (95% CI)	Workout consistent users (n=618), AOR (95% CI)
**Sex (reference group=male)**						
	Female	2.09^c^ (1.27-3.44)	2.13^c^ (1.26-3.60)	1.62 (0.91-2.91)	—^d^	—
**Generation (reference group=generation Y)**						
	Baby boomers	2.54^c^ (1.31-4.92)	2.02^c^ (1.04-3.92)	2.51^c^ (1.27-4.93)	3.06^c^ (1.56-6.02)	3.21^c^ (1.62-6.36)
	Generation X	1.96^c^ (1.42-2.72)	1.86^c^ (1.34-2.59)	1.92^c^ (1.36-2.71)	1.59^c^ (1.12-2.25)	2.05^c^ (1.38-3.05)
	Generation Z	0.77 (0.44-1.35)	0.69 (0.39-1.23)	0.69 (0.35-1.34)	0.52 (0.26-1.05)	0.28^c^ (0.08-0.94)
**Group size (reference group=small: 2-5 members per group)**						
	Individual (1 member per group)	0.24^c^ (0.07-0.84)	0.21^c^ (0.05-0.94)	0.75 (0.20-2.86)	0.18 (0.02-1.47)	0.25 (0.03-1.98)
	Medium (6-10 members per group)	2.06^c^ (1.47-2.88)	2.30^c^ (1.63-3.24)	2.51^c^ (1.72-3.66)	1.74^c^ (1.20-2.53)	1.57^c^ (1.02-2.42)
	Large (≥11 members per group)	2.85^c^ (1.58-5.16)	3.55^c^ (1.92-6.55)	4.19^c^ (2.20-7.98)	2.88^c^ (1.50-5.52)	2.29^c^ (1.13-4.64)

^a^Variables in the table remained after model adjustment.

^b^AOR: adjustable odds ratio.

^c^*P*<.05.

^d^Not applicable.

## Discussion

### Principal Results and Comparison With Previous Work

The MED PSU×ThaiSook challenge participants were primarily female, belonged to generation Y, and had a normal BMI. Most participants were in the medium-sized group, followed by small-sized and large-sized groups (in that order). Female participants used the app more frequently than male participants. Compared to individual usage, group participation was associated with higher adherence. The older generation participated more routinely than did the younger generation. In contrast, the initial BMI did not significantly affect user participation. The most regularly used logging function was water logging, followed by fruit and vegetable logging, sleep logging, and workout logging. Overall, app usage was significantly higher in women, older generations, and larger groups than in men, younger generations, and smaller groups, respectively. However, it is important to acknowledge that multiple factors influence health outcomes, including genetics, environmental factors, socioeconomic status, and health care access. While recording health data through mHealth apps can contribute to better health management, it is only 1 aspect of a comprehensive approach to health care.

Our findings indicated that female participants were more likely to be consistent users than male participants. The results of this study on sex corresponded with the findings of previous studies: compared to men, women were more likely to think about well-being and friendships [21]. In addition, the sample comprised predominantly females.

The ThaiSook app was designed to be user friendly, intuitive, easy to access, and simple to navigate, catering to users of all generations. A key feature of the app is its streamlined login process, requiring users to log in only once using a 1-time password sent to their phone number. The interface for logging activities has been carefully crafted to effectively fulfill its intended purpose. In this study, app usage was higher in the older generations than in the younger generations in a dose-response relationship (biological gradient) as per Bradford Hill's criteria for causation in Epidemiology [30]. Surveys and previous studies have shown that younger generations are more interested in technology and are more likely to use health apps and other tracking devices (such as smartwatches). Older generations were expected to have lower adoption rates due to less familiarity with technology, while younger generations were expected to show more openness toward using the mHealth app. Our results contradict this hypothesis. Considering the usability design of the ThaiSook app, we hypothesize that the ease of use significantly influences the engagement of older generations with the mobile health app. These results could indicate that if the app is designed to be easily accessible and intuitive, it may foster greater usage consistency among older users. By prioritizing usability in the design process, the app can effectively cater to the needs and preferences of older generations, encouraging sustained engagement and use. These generations also tend to remain loyal to the app, whereas younger generations may discontinue or consider using other health apps because of their higher performance expectations.

The effect that grouping has on establishing more power in an individual’s development and behavior is known as the “peer effect” [22]. German sociologist Georg Simmel discovered that larger groups create more stability for survival compared to a member leaving the group. However, the larger a group becomes, the more its members are separated from each other and the more they develop independence [22]. This study found that participants in large groups used the app most regularly, corresponding to the peer effect and Simmel’s observations. Large groups were more stable even though some members could be less active in using the app; the more active users influenced others to stay consistent, leading to a higher number of consistent and daily users overall. Nevertheless, the impact of larger groups on behavioral changes and health outcomes, such as weight loss, requires further study.

Water logging was the most frequently used logging function, which can be explained by the fact that participants could record a maximum amount of 200 mL for each log. For example, 400 mL of drinking water were considered 2 logs of 200 mL and were counted as 2 records. In addition, water logging is easy to use compared to the other logging functions. For example, the participants did not upload details on fruit and vegetable and workout logging as frequently as they did for water logging (details in Table 1). Therefore, the study results indicated that water logging had a higher daily usage frequency compared to other logging features. Following closely was fruit and vegetable logging, likely due to the 3 daily meals that most people consume. Workout logging, on the other hand, had the lowest usage frequency as daily exercise was less common among participants.

### Strengths and Limitations

The strength of this study lies in its novelty: it is among the first to examine the association between generations, group sizes, and mHealth app use. This study is only the second to collect real-world data on mHealth app usage in an upper- to middle-income country [31]. ThaiSook is an app developed by a national organization with standards established to ensure quality.

This study has some limitations. First, the response rate for the MED PSU×ThaiSook Challenge was 13.5%. The selection bias should be acknowledged in this study. The participants might be more interested in health promotion than those who did not participate in the challenge. Second, the MED PSU×ThaiSook Challenge was a competition with team-based rewards; both the competition and the rewards drove enthusiasm in participant usage; thus, the data may be different from normal usage data. Third, the participation period may have been too short to observe long-term app usage. People may tend to use the app more consistently during the early phase. According to the Transtheoretical Model, each stage takes more than a month to eventually build a routine use of the app [32]. Fourth, there is a lack of generalizability owing to the limited population and specific situations in the challenge. This study’s sample comprised health care personnel with more health care knowledge, which could influence personal awareness and concerns [33]. Finally, incomplete self-reported data (65/827, 7.9%) could also impact this study's accuracy.

### Implication and Further Studies

Our results suggest that group use should be given greater consideration when developing new mHealth apps. This study showed that a team-based model produces a peer effect, for example, more group members tend to create more consistent users. In addition, design and functions should focus on target users who are females, baby boomers, and belong to generation X. Additionally, mHealth apps that are free to download can benefit anyone interested in a healthy lifestyle. For future research, we recommend extending the observation period to assess long-term behavioral changes and conducting the challenge in varied settings to examine its effectiveness in different contexts.

### Conclusions

The MED PSU×ThaiSook Healthier Challenge participants were predominantly female, from generation Y, and from medium-sized organizations. The most frequently used logging function was water logging, followed by fruit and vegetable logging. According to the findings, generation and group size were substantially related to consistent and everyday usage. Surprisingly, older generations used the app more than younger generations. Furthermore, larger groups used the app with greater consistency than smaller groups and individuals. This study suggests that group use should be considered when developing new mHealth apps, as it leads to more consistent users. Targeting females, baby boomers, and generation X is important. Free downloads benefit those interested in a healthy lifestyle.

## Abbreviations

AOR: adjusted odds ratio
MED PSU: Faculty of Medicine, Prince of Songkla University
mHealth: mobile health
NSTDA: National Science and Technology Development Agency
PSU: Prince of Songkla University

## Data Availability

The data sets used and analyzed during this study are available from the corresponding author upon reasonable request.
